# Multifunctional Electrospun Nanofibers Based on Biopolymer Blends and Magnetic Tubular Halloysite for Medical Applications

**DOI:** 10.3390/polym13223870

**Published:** 2021-11-09

**Authors:** Viera Khunová, David Pavliňák, Ivo Šafařík, Martin Škrátek, František Ondreáš

**Affiliations:** 1 Faculty of Food and Chemical Technology, Institute of Natural and Synthetic Polymers, Slovak University of Technology, Radlinského 9, 812 37 Bratislava, Slovakia; 2 CEPLANT, Department of Physical Electronics, Masaryk University, Kotlářská 267/2, 611 37 Brno, Czech Republic; d.pavlinak@mail.muni.cz; 3 Department of Nanobiotechnology, Biology Centre, ISB, CAS, Na Sádkách 7, 370 05 České Budějovice, Czech Republic; ivosaf@yahoo.com; 4 Regional Centre of Advanced Technologies and Materials, Czech Advanced Technology and Research Institute, Palacký University, Šlechtitelů 27, 783 71 Olomouc, Czech Republic; 5 Institute of Measurement Science, Slovak Academy of Sciences, Dúbravská Cesta 9, 841 04 Bratislava, Slovakia; Martin.Skratek@savba.sk; 6 CEITEC—Central European Institute of Technology, Brno University of Technology, Purkyňova 123, 612 00 Brno, Czech Republic; frantisek.ondreas@ceitec.vutbr.cz; 7 CONTIPRO a.s., Dolní Dobrouč 401, 561 02 Dolní Dobrouč, Czech Republic

**Keywords:** magnetic, halloysite, nanotubes, nanofibers, biopolymer, polycaprolactone, gelatine, electrospinning

## Abstract

Tubular halloysite (HNT) is a naturally occurring aluminosilicate clay with a unique combination of natural availability, good biocompatibility, high mechanical strength, and functionality. This study explored the effects of magnetically responsive halloysite (MHNT) on the structure, morphology, chemical composition, and magnetic and mechanical properties of electrospun nanofibers based on polycaprolactone (PCL) and gelatine (Gel) blends. MHNT was prepared via a simple modification of HNT with a perchloric-acid-stabilized magnetic fluid–methanol mixture. PCL/Gel nanofibers containing 6, 9, and 12 wt.% HNT and MHNT were prepared via an electrospinning process, respecting the essential rules for medical applications. The structure and properties of the prepared nanofibers were studied using infrared spectroscopy (ATR-FTIR) and electron microscopy (SEM, STEM) along with energy-dispersive X-ray spectroscopy (EDX), magnetometry, and mechanical analysis. It was found that the incorporation of the studied concentrations of MHNT into PCL/Gel nanofibers led to soft magnetic biocompatible materials with a saturation magnetization of 0.67 emu/g and coercivity of 15 Oe for nanofibers with 12 wt.% MHNT. Moreover, by applying both HNT and MHNT, an improvement of the nanofibers structure was observed, together with strong reinforcing effects. The greatest improvement was observed for nanofibers containing 9 wt.% MHNT when increases in tensile strength reached more than two-fold and the elongation at break reached a five-fold improvement.

## 1. Introduction

Magnetically responsive nano- and microparticles and high aspect ratio materials and films have found many important applications in various areas of biosciences, medicine, (bio)analytical chemistry, biotechnology, and environmental technology. These magnetic (nano)materials exhibit several types of responses to external magnetic fields, including selective separation, targeting or localization, heat generation in high-frequency alternating magnetic fields, increases in negative T2 contrast during magnetic resonance imaging, and increases in apparent viscosity of magnetorheological fluids when subjected to a magnetic field [1,2,3,4,5]. Both free and bound magnetite nanoparticles also exhibit peroxidase-like activity [6,7,8].

Diamagnetic materials including adsorbents, catalysts, chromatography materials, carriers, microbial cells, (nano)textile, and biological waste materials can be converted into magnetically responsive derivatives using various modification procedures in order to improve their application potential. Magnetic modification usually leads to the formation of strongly magnetic composite materials, whereby the ‘‘original’’ diamagnetic structure is responsible for the biological, catalytic, carrier, or adsorption function of the formed composite, while the magnetic label (most often in the form of magnetic iron oxide nano- and microparticles, which are usually deposited on the surface or within the pores of treated materials) is responsible for the strong magnetic behavior of the formed composite materials [9].

The ideal procedure for magnetic modification of diamagnetic materials should be inexpensive, easy to perform, scalable, and tuneable, leading to a stable magnetic product, both in dry state and water suspension [10]. Gentle modification procedures are based on the deposition of magnetic iron oxide or ferrite nano- or microparticles on the surfaces or within the pores of the treated biomaterials, e.g., using an appropriate magnetic fluid [11]. Additionally, microwave-synthesized magnetite nano- and microparticles prepared from ferrous sulfate at high pH have been applied for magnetic modification; the stable magnetic particle binding was caused by the final heat treatment [12] or by long-term freezing at temperatures below −20 °C [13].

Electrospun nanofibers and nanotextiles represent a progressive group of high aspect ratio nanomaterials with many interesting potential medical applications. They can be used for long-term continuous drug delivery for potential oncological applications [14]; as drug-loaded electrospun materials in wound-dressing applications [15]; or as carriers of different non-steroidal anti-inflammatory drugs such as ketoprofen [16], flurbiprofen axetil [17], and ibuprofen [18]. More information can be found in a recently published review paper [19].

Additionally, nanotextile materials can be prepared in magnetic form and four basic procedures can be used. Surface-stabilized magnetic iron oxide nanoparticles are usually directly mixed with the polymer solution and electrospinning is performed; to obtain homogeneous nanoparticle dispersions in a mixture, the mechanical stirring and ultrasonication are usually employed [20,21]. In another approach (in situ synthesis), a polymer solution containing FeCl_3_ and FeSO_4_ was employed; electrospinning was carried out in an ammonia atmosphere to convert iron compounds to iron oxide nanoparticles [22]. Alternatively, already prepared nanotextiles can be magnetically modified by immersion into magnetic fluid or by spraying [23]. In addition, the simple post-magnetization process can be employed, involving the immersion of the produced nanotextile in an aqueous solution containing Fe (II) and Fe (III) salts followed by the addition of a weak base to yield magnetic iron oxide nanoparticles [24]. A recent review describing the preparation, properties, and applications of electrospun magnetic nanomaterials is available [25].

Magnetically modified nanotextiles have been used as scaffolds for better osteoblast growth [26]. Superparamagnetic electrospun microrods were also prepared for magnetically guided pulmonary drug delivery with magnetic heating [27]. A biocompatible electrospun nanotextile heavily loaded with magnetic iron oxide nanoparticles was suggested recently for magnetic hyperthermia treatment [28,29].

An increasing number of studies are focusing on the creation of magnetically responsive scaffolds for tissue engineering. Nanoparticles in a biomaterial substrate are exposed to an external magnetic field, which leads to local deformation of the substrate, which is able to activate a mechanotransduction mechanism on the cells. Such magnetic stimulation strategies have shown positive effects in tendon [30,31], bone [32,33], cardiac [34], vascular, and tissue engineering (TE) [35].

Superparamagnetic iron oxide nanoparticles have many excellent characteristics, such as non-toxicity, good biocompatibility, and unique magnetic targeting properties; in addition, they can be prepared using a wide variety of procedures. However, from a magnetic point of view, nanoparticles exhibit specific magnetic properties that differ from those of the bulk with the same chemical composition. As a result, the magnetic properties of polymer nanocomposites depend first of all on the magnetic nanofiller’s particle size and shape, morphology, concentration, and distribution in the polymer matrix. It was found that by reducing the size of the nanoparticles from tens to a few nanometers, the magnetization of the single nanoparticles was no longer stable and the saturation magnetization decreased rapidly [36].

Electrospun (nano)fibers and (nano)textile can also be modified via the entrapment of appropriate nanomaterials. One of the most progressive examples of this approach is tubular halloysite (HNT), a naturally occurring, two-layered 1:1 phyllosilicate clay. HNT has a similar structure to kaolin clay, except that it contains additional water molecules between the layers [37,38,39]. One of the important features of halloysite is its different surface chemistries at the inner and outer sides of the tubes; silica sheets correspond to the external surfaces of the tubes, while aluminium oxide makes up the inner (lumen) surface chemistry [40]. HNT present a unique combination of natural availability, good biocompatibility, non-toxicity, high mechanical strength, and functionality. Moreover, due to its rod-like morphology and large aspect ratio, the application of HNT in nanocomposites and nanofibers has a strong reinforcing effect [41,42,43]. For potential biomedical applications, it is very important that HNT can be used as a low-cost nanocontainer for the encapsulation of a variety of different chemically and biologically active substances, including drugs, enzymes, DNA, and many others into the inner or outer surfaces of nanotubes [44,45,46,47]. In addition, by depositing metals or metal oxides on external or interior HNT walls, diamagnetic HNT can be easily transformed into magnetically responsive material.

There are several techniques that can be used for the magnetic modification of diamagnetic materials, including HNT. In most cases, the magnetization of HNT is based on the attachment of magnetic iron oxide nano- or microparticles on the surface or within the pores of the HNT [11,48].

Recently, magnetically modified HNT was prepared using mechanochemical synthesis; HNT with the deposited magnetic iron oxide nanoparticles and their aggregates were successfully applied for the removal of silver nanoparticles from a water dispersion [49]. Alternatively, microwave-synthesized magnetite nano- and microparticles from ferrous sulfate at high pH [12] have been used to prepare magnetic HNT to study their effects on the structure and properties of biodegradable polymer nanocomposites based on poly ϵ-caprolactone (PCL) [50] or polycaprolactone-gelatine nanofibers [51]. Additionally, coprecipitation of magnetic iron oxide particles from iron (II) and iron (III) salts in the presence of HNT has been employed successfully [52,53]. Alternatively, oleic-acid-stabilized magnetic fluid was used to modify the inner lumen of HNTs modified by tetradecylphosphonic acid [54]. The magnetic properties of magnetized halloysite (MHNT) depend on the type of magnetic (nano)particles bound to the tube surfaces; modification with magnetic nanoparticles usually leads to the formation of superparamagnetic materials [55].

In recent years, a number of techniques for the preparation of submicron fibers have been developed and improved, such as melt spinning, centrifugal spinning, and pressurized gyration spinning; each has its own advantages and disadvantages [56,57,58,59]. At present, the electrospinning method is the most widespread method worldwide for the preparation of nanofibers with unique properties [60]. Despite its energy consumption and sensitivity to the quality of the input polymer solution, this technique allows for very simple implementation in industrial-scale production [61,62]. Based on our previous experience, we chose the free-liquid spinning method, which is very close to the processes used in industrial practice [61,62]. For this reason, instead of conventional laboratory spinning units, we worked on a semi-industrial NSLSAB 500 device (Elmarco, Liberec, Czech Republic), which allows the production of nanofiber membranes in continuous in-line mode up to a winding width of 50 cm. As a polymer matrix, a blend of hydrophobic polycaprolactone (PCL) and hydrophilic gelatine (Gel) was used, which are highly desirable for medical applications [63]. In this study, we explored the effects of MHNT on the structure and mechanical and magnetic properties of amphiphilic PCL/Gel nanofibers. Respecting principles of “green chemistry” required for medical applications, acetic acid was used as the solvent. For magnetic modification, a very simple alternative procedure employing a perchloric-acid-stabilized magnetic fluid-methanol mixture was employed [64]. Repetition of this modification procedure led to higher deposition rates of magnetic nanoparticles on the halloysite surfaces.

## 2. Materials and Methods

### 2.1. Materials

Polycaprolactone (PCL − Mn = 80 kDa), gelatine (Gel–Type B, bovine skin) and halloysite (HNT, cat. No. 685445) were bought from Sigma-Aldrich (St. Louis, MO, USA). Acetic acid (99% p.a.) was purchased from Penta s.r.o, Prague, Czech Republic. All compounds were used as received without further purification.

### 2.2. Methods

#### 2.2.1. Magnetic Modification of Halloysite

The perchloric-acid-stabilized magnetic fluid (ferrofluid) was prepared using the standard procedure [65]. The ferrofluid was mostly composed of magnetic iron oxide nanoparticles with diameters ranging between 5 and 20 nm (electron microscopy measurements); the relative magnetic fluid concentration (30.8 mg/mL) is given as the iron (II, III) oxide content determined using a colorimetric method [66]. Magnetic modification of halloysite was performed in the following way. Halloysite (1 g) was suspended in 30 mL of methanol in a 50 mL plastic centrifugation tube, then 2 mL of acid ferrofluid was added and the suspension was thoroughly mixed on the rotating mixer for 2 h. Then, the ferrofluid modified halloysite was separated using a magnetic separator, the methanol–ferrofluid supernatant was removed, then 30 mL of fresh methanol and 2 mL of ferrofluid were added and mixing on the rotating mixture continued for another 2 h. This procedure was repeated twice more. At the end, the modified halloysite was repeatedly washed with methanol using a magnetic separator. The washed magnetically modified halloysite was dried at 50–60 °C for 24 h.

#### 2.2.2. Preparation of PCL/Gel Solutions

The PCL-gel solution was prepared by adding a calculated amount (weight ratio 1:1) of PCL and gel into acetic acid to form a 12 wt.% polymer solution. PCL/Gel/HNTs and PCL/Gel/MHNTs mixtures were obtained by adding 6, 9 and 12 wt.% of HNTs or MHNTs to the PCL/Gel solution. Then, the mixtures were stirred overnight.

#### 2.2.3. Electrospinning Process

Prior to the electrospinning process itself, the mixtures were dispersed in an ultrasound bath for 15 min. Electrospun mats were prepared using an NS LAB 500S laboratory machine from Elmarco s.r.o. (Liberec, Czech Republic) at room temperature (approx. 25 °C) and a relative humidity of 40%. The applied voltage range was 60–70 kV.

### 2.3. Characterization

#### 2.3.1. Structure and Composition of Raw and Magnetic Halloysite

This process was performed using ATR-FTIR spectroscopy (Bruker VERTEX 80V, BRUKER, Leipzig, Germany) and SEM+EDX (STEM) (MIRA 3, TESCAN, Brno, Czech Republic). The morphology of the nanofibers was investigated using a scanning electron microscope (SEM, STEM). The fiber diameters were measured using Image J software Version 1.5 (National Institute of Health, Bethesda, MD, USA).

#### 2.3.2. Magnetic Properties

The magnetic properties were measured using a SQUID Quantum Design MPMS XL-7T magnetometer (Quantum Design, San Diego, CA, USA). Characterization of the magnetized HNT and PCL/Gel/MHNT was done via measurement of the temperature dependence of the magnetization. This was measured in two ways: first the sample was cooled from room temperature (RT, 300 K) down to 1.8 K without applying any magnetic field (using ultra low field option to set zero field) and then heated back to RT under an applied magnetic field of 50 Oe (zero field cooled—ZFC curve); afterwards, under the same field, the sample was cooled down again to 1.8 K, following the same steps as before (field cooled—FC curve). Additionally, measurement of the magnetization as a function of the field (M(H) curve) was done at 2 and 300 K with an applied magnetic field of up to 7 T. For characterization of the prepared nanofibers, only M(H) curves at 2 and 300 K were measured.

#### 2.3.3. Mechanical Properties

The mechanical properties were tested under uniaxial tension at a crosshead speed of 5 mm·min^−1^ and ambient temperature of 22 °C using a Zwick Roell Z010 (Zwick-Roell, Ulm, Germany) equipped with a 10 N load cell. Six rectangular specimens (approximately 30 × 5 mm) were tested for each type of material and the average and standard deviation were determined. The thickness of each specimen was measured with a micrometer and ranged between approximately 0.1 and 0.4 mm.

## 3. Results and Discussion

### 3.1. Structure and Composition of Magnetized Halloysite (MHNT)

The magnetic modification of diamagnetic powders and high aspect ratio materials enables the preparation of composites with a broad range of applications. Several procedures have already been applied by our research team for magnetic modification of HNT, either by applying the mechanochemical treatment [49] or via modification with microwave-synthesized magnetic nanoparticles [50]. A very simple alternative procedure employing perchloric an acid-stabilized magnetic fluid/methanol mixture [11,64] was employed for magnetic modification of HNT in this report.

**Figure 1:**
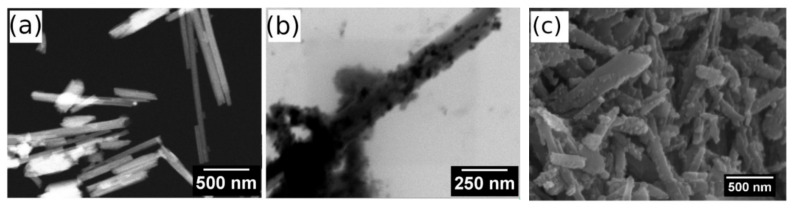
STEM images of (**a**) raw halloysite (STEM dark mode, magnification 100,000×) and (**b**) magnetized halloysite (STEM bright mode, magnification 200,000×; the magnetic particles are represented by black dots). (**c**) SEM image of magnetically modified halloysite.

To evaluate the structure and composition of unmodified halloysite (HNT) and magnetic halloysite (MHNT), the samples were analyzed using both the classical method of scanning electron microscopy (SEM) supplemented with elemental analysis (EDX) and in scanning transmission electron microscopy (STEM) mode. Figure 1 shows halloysite particles taken in STEM mode. Although the resolution of the images does not reach the quality obtained with commercial transmission microscopes (TEM), it is clear that the particles of the unmodified halloysite are tubular structures.

The HNT particles measured 944 to 1640 nm in length and 67 to 99 nm in diameter (Figure 1a). After chemical treatment and grafting of the magnetic nanoparticles (Figure 1b,c), the aspect ratio changed very little; however, the surfaces of the HNT particles became bumpy and were almost entirely covered with magnetic nanoparticles. The MHNT particles measured 1022 to 1668 nm in length and 77 to 139 nm in diameter, while the magnetic nanoparticles measured 5 to 20 nm in diameter (with most measuring approximately 10 nm in diameter).

**Figure 2:**
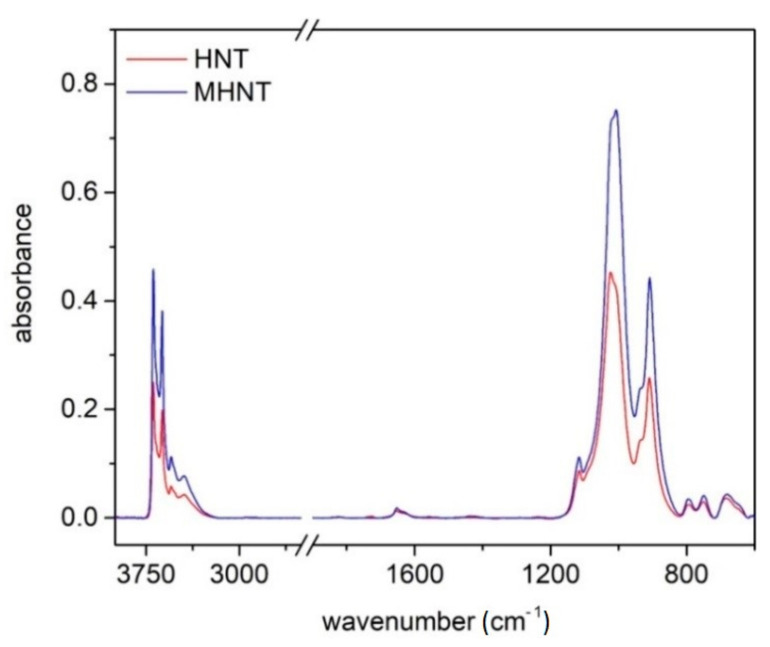
FTIR spectra of HNT and MHNT powder.

Figure 2 shows FTIR spectra of HNT and MHNT. The double peaks at 3692 and 3622 cm^−1^ are caused by stretching vibrations associated with the HNTs’ surface hydroxyl groups [55,67]. Similarly, the intensive band at 908 cm^−1^ confirms the presence of internally bonded OH groups (Al–OH) via their deformation vibration. The vibrations near 1000 cm^−1^ can be assigned to Si–O stretching (1116 cm^−1^) or Si–O–Si bonds (1024 and 1006 cm^−1^). In addition, the bands at 794 cm^−1^ and 754 cm^−1^ correspond to the out of plane and Si–O–Al vibrations [68]. The vibrations at 1650–1630 cm^−1^ are interesting, which indicate the presence of interlayer water molecules [67]. Other IR bands indicating the presence of Al–O–Si bonds (ca. 530 cm^−1^) and magnetic particles (ca. 580 cm^−1^) are not visible due to the limitations of the measured spectral range.

### 3.2. Structure and Composition of PCL/Gel/MHNT Nanofibers

**Figure 3:**
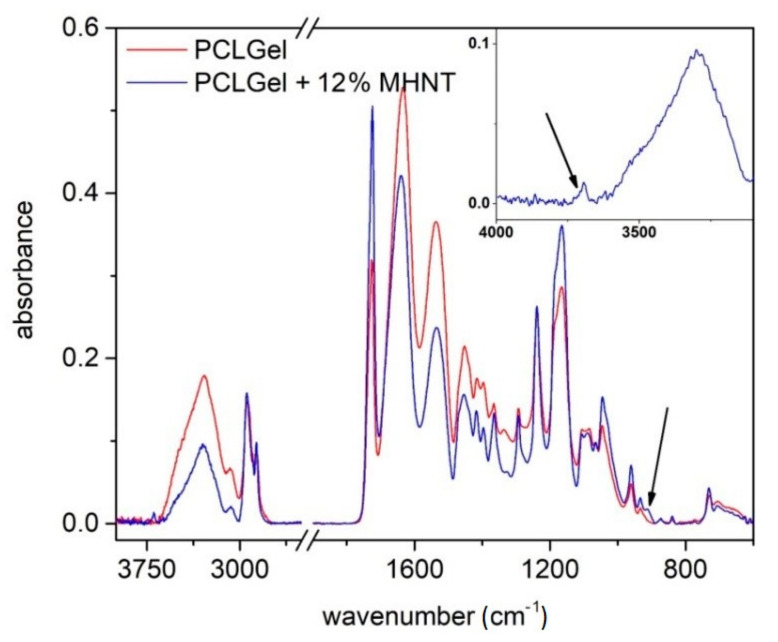
FTIR spectra of PCL/Gel nanofibers doped by magnetized HNT particles. The OH vibrations originating in MHNTs are highlighted by arrows.

In Figure 3, the FTIR spectrum of PCL/Gel nanofibers doped by magnetically modified HNT particles (MHNT) is shown. Because HNT particles are present in the nanofibers at a relatively small concentration, they will make a minimal contribution, and vibrations originating from polymer matrix predominate in the overall IR spectrum.

The PCL/Gel spectrum presented in the absorption bands is characteristic of gelatine (3279 cm^−1^ amide A, 1640 cm^−1^ amide I, 1538 cm^−1^ amide II, and 1240 cm^−1^ amide III), as well as for polycaprolactone (2937 cm^−1^ asymmetric CH_2_ stretching, 2867 cm^−1^ symmetric CH_2_ stretching, 1722 cm^−1^ carbonyl stretching, 1241 cm^−1^ O–C–O stretching, 1168 cm^−1^ symmetric C–O–C stretching, and 1028 cm^−1^ bridging oxygen vibrations).

Vibrations of MHNT particles are mainly overlapped by the polymer matrix; only OH vibrations at 3692 and 908 cm^−1^ are visible. If these spectra are compared with our previous published experiments [68], where we added non-magnetized HNT to the same PCL/Gel solutions, it may seem that the intensity of HNT vibrations and the concentration of halloysite in the fibers are lower in the case of magnetized HNT. This may be due to the different methods of mixing and dispersing the particles in solution, as the usual procedure is to stir the non-magnetized HNT in the polymer solution on a magnetic stirrer overnight. In our case, a (non-magnetic) homogenizer had to be used to ensure sufficient particle dispersion. However, for practical reasons, the dispersion time was reduced to a few minutes. This raises the question of whether larger agglomerates of MHNT particles formed in the solution when the agitation process changed. Large agglomerates have difficulty entering the spinning process and do not pass into the nanofibers, which in turn can lead to a decreased concentration of MHNT particles in the fibers. Although the presence of magnetized HNT in PCL/Gel nanofibers has already been well demonstrated by infrared spectroscopy, showing the extent and homogeneity of the magnetized particle distribution in the nanofibers, we decided to perform an electron microscopy analysis.

**Figure 4:**
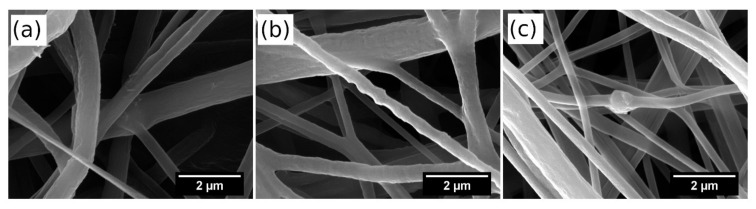
SEM images of PCL/Gel nanofibers doped with (**a**) 6 wt.%, (**b**) 9 wt.%, and (**c**) 12 wt.% HNT.

**Figure 5:**
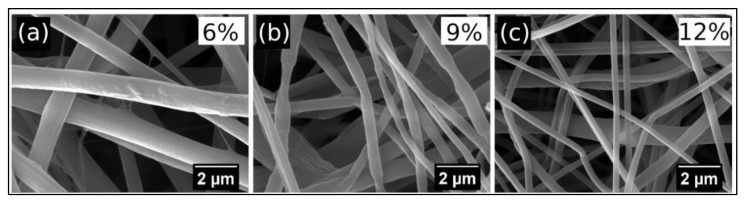
SEM images of PCL/Gel nanofibers doped with 6 wt.%, (**a**), 9 wt.% (**b**), and 12 wt.% (**c**) magnetized halloysite. Magnification = 25,000×.

Figure 4 shows the morphology of the prepared nanofibers containing unmodified halloysite at concentrations of 6 wt.%, 9 wt.%, and 12 wt.%. The morphology of the nanofibers comprising the same contents of magnetized halloysite MHNT is presented in Figure 5. It can be seen that the diameter of the fibers decreases with increases in the contents of unmodified and magnetized halloysite. MHNT particles that are not fully dispersed in the original polymer solution will subsequently form clumps of particles, contributing to the formation of a bead structure in the fibers.

**Figure 6:**
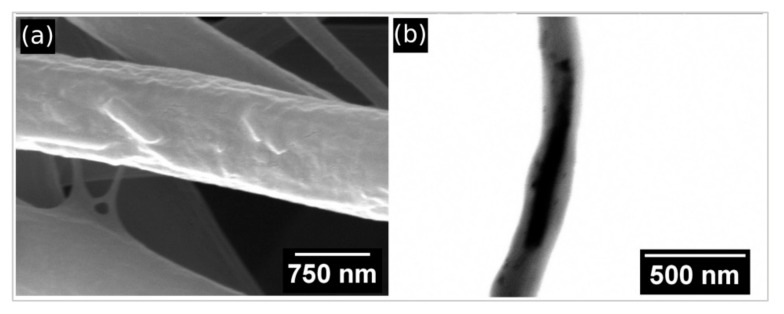
(**a**) Detailed SEM image of PCL/Gel nanofibers doped with 6 wt.% MHNT, with visible MHNT particles on the surface (magnification = 75,000×). (**b**) STEM image (bright mode) of MHNT particles inside the PCL/Gel nanofibers (magnification = 150,000×).

Detailed observation of a single fiber from Figure 5a revealed that MHNT particles are visible on the surfaces of PCL/Gel nanofibers (Figure 6a); however, in most cases MHNT particles are localized inside the nanofibers (Figure 6b).

Table 1 shows the element contents of HNT, MHNT, and PCL/Gel/MHNT, as measured by EDX microanalysis. There is a quantitative increase in the iron content (up to 8 wt.%) after grafting of magnetic nanoparticles on the surface of HNT. However, in the nanofiber material itself, the content of elements characteristic for MHNT particles is very low, probably due to the dilution of MHNT in the polymer matrix. When focusing on beaded structures (where a higher occurrence of MHNT particles can be expected) or particles visible on nanofibers surface (e.g., Figure 5a), the Fe content increases up to 2 wt.%.

The content of elements characteristic of PCL/Gel/MHNT was below 1 wt.% This is at the limit of the sensitivity of the EDX determination and the values can only be considered as indicative. For this reason, the values for 6–12 wt.% MHNT are given together (Table 1), although increases in the contents of Al, Si and Fe were observed with increasing concentration of MHNT in the sample. These values were averaged and taken at low magnification analysed from the entire image area. When focusing on beaded structures (where a higher occurrence of MHNT particles can be expected), the Fe content increased up to 2 wt.%.

### 3.3. Mechanical Properties of PCL/Gel/MHNT Nanofibers

**Figure 7:**
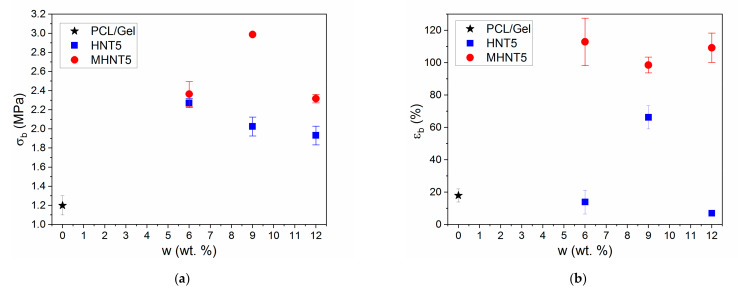
Tensile strength (**a**) and elongation at break (**b**) results for PCL/Gel nanofibers with HNT and MHNT particles at various concentrations.

As already mentioned, the application of HNT in nanocomposites and nanofibers can have a strong reinforcing effect [41,42,43]. However, the reinforcing effect depends on the type of polymer matrix, HNT aspect ratio and dispersion in the polymer matrix. In this study, it was found that the incorporation of both HNT and MHNT particles into PCL/Gel nanofibers led to a more than two-fold increase in tensile strength (Figure 7a). The maximum improvement was observed for PCL/Gel/MHNT with 9 wt.% of particles. Furthermore, a five-fold increase in the elongation at break was observed for the PCL/Gel/MHNT samples compared to the neat PCL/Gel nanofibers (Figure 7b).

The increase in the elongation at break was independent of the MHNT weight fraction. A similar complex dependence of the mechanical properties on the nanoparticle (NP) concentration was described for bulk systems [63,68,69,70,71]. The observed pronounced increase in mechanical properties was caused by multiple reinforcing mechanisms. The HNT showed much higher stiffness than the PCL/Gel matrix and a high aspect ratio. Therefore, volume replacement and stress transfer mechanisms were active. Furthermore, the dimensions at nanometer-scale and the large interface area led to the significant immobilization of the polymer chain onto NP surfaces. This led to the creation of the modified polymer matrix with enhanced mechanical performance. The initial sharp increase in properties with the NPs fraction was caused mainly by activation of this mechanism. However, further increases in the NP weight fraction could lead to the interparticle distance being decreased below the modified polymer layer thickness and possibly also to the slight worsening of the dispersion state. Therefore, the immobilization did not contribute to the improvement of the mechanical properties, which stagnated or decreased with increased NP weight fraction above a certain value. The surface area and polymer–particle interaction strength can control the response of polymer nanocomposites [71,72,73,74,75]. The presence of magnetic NPs on the HNT increased the interfacial area and improved polymer-particle interactions, as superior mechanical performance was observed for the nanofibers reinforced by MHNT against HNT. Moreover, the modification of the NP surface interactions can also play an important role during wet processing when particles interact with solvent molecules [72,73,76]. Therefore, the surface modification could improve the dispersion of MHNT particles and solution properties influencing processability via electrospinning.

### 3.4. Magnetic Properties of PCL/Gel/MHNT Nanofibers

**Figure 8:**
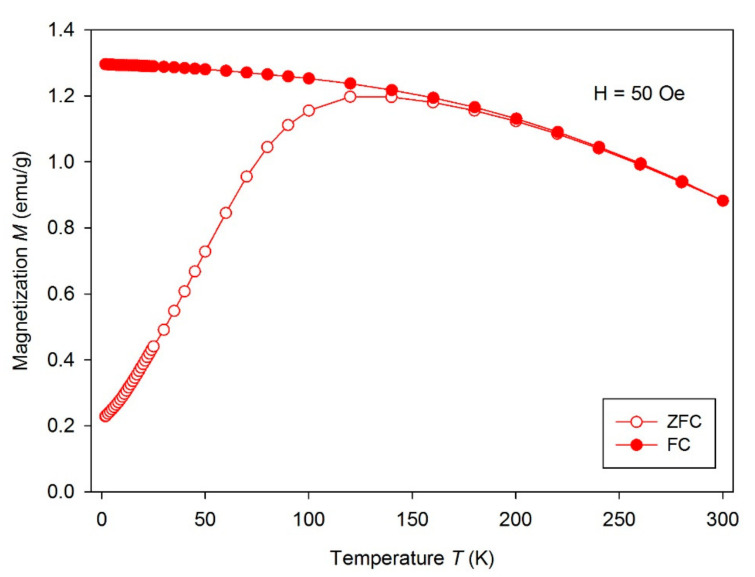
Temperature dependence of magnetization measured for MHNT.

**Figure 9:**
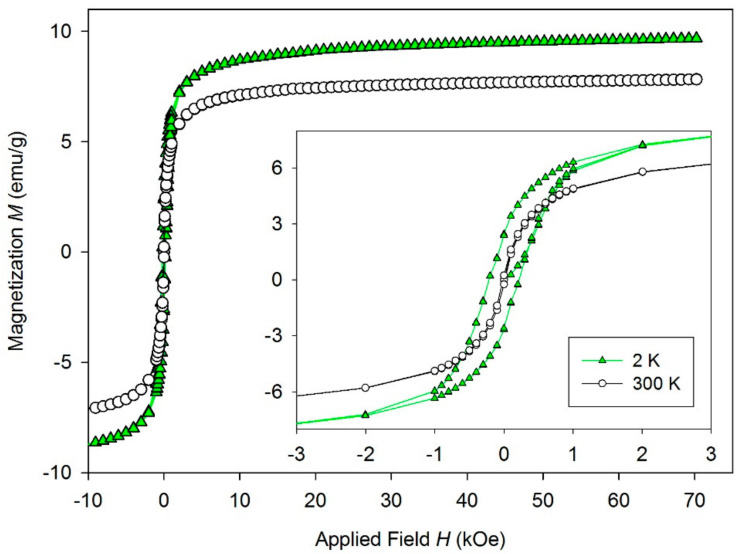
Isothermal hysteresis loops measured at 2 and 300 K for MHNTs. Inset represents low field part of the hysteresis loops.

The measured temperature dependence of the magnetization (Figure 8) of MHNT showed bifurcation between ZFC and FC curves and a blocking temperature *T*_B_ of 120 K. This is a sign of superparamagnetic properties of nanoparticles being present on the surface of MHNT. According to the SEM examination, the magnetic nanoparticles were not isolated from each other and were bound to HNT, meaning the *T*_B_ was higher than expected for such small nanoparticles. The *M*(*H*) dependence (Figure 9) measured at 2 K showed slight hysteresis (inset of Figure 9), although the presence of hysteresis is temperature-dependent and over *T*_B_ it disappears. The dependence measured at 300 K showed almost no hysteresis, with saturation magnetization *M*_S_ = 7.8 emu/g, which is characteristic for superparamagnetic nanoparticles. A summary of the magnetic parameters *M*_S_, remnant magnetization *M*_R_, coercivity *H*_C_, and *T*_B_ is given in Table 2.

**Figure 10:**
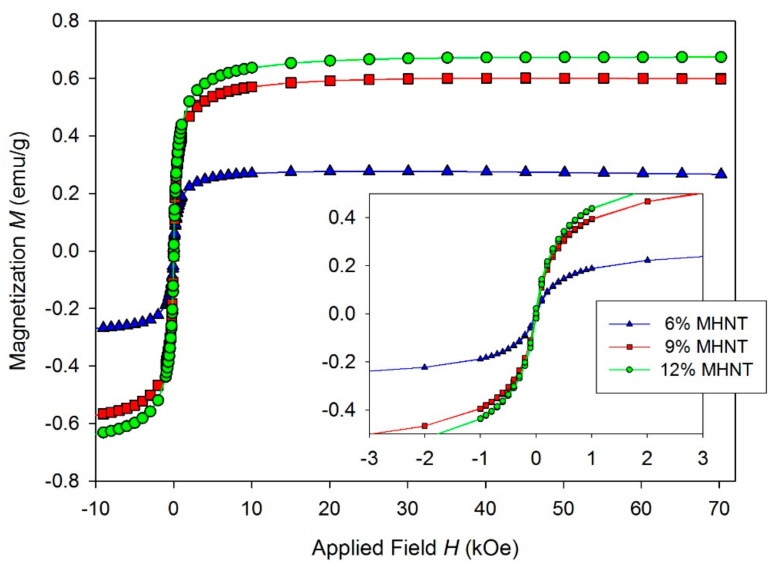
Isothermal hysteresis loops of PCL/Gel with 6.0 wt.% MHNT (triangle), PCL/Gel with 9.0 wt.% MHNT (square), and PCL/Gel with 12.0 wt.% MHNT (circle). Inset is the low field part of the loops.

The measured *M*(*H*) curves of the studied PCL/Gel/MHNT nanofibers (Figure 10) confirmed that the nanofibers retained properties of the MHNT with the mass magnetization proportional to the percentual weight content of MHNT. The low field part of the *M*(*H*) curves (inset on Figure 10) again showed almost no hysteresis for PCL/Gel/MHNT nanofibers.

The low coercivity H_C_, remnant magnetization M_R_, and distinct hysteresis loops confirm the superparamagnetic behavior of the PCL/Gel/MHNT electrospun nanofibers (Table 2).

## 4. Conclusions

In this study, electrospun PCL/Gel nanofibers reinforced by 6, 9, and 12 wt.% of raw halloysite (HNT) and magnetically modified halloysite (MHNT) have been studied. For the magnetic modification of halloysite, a perchloric-acid-stabilized magnetic fluid/methanol mixture was employed. Due to the very small (5–20 nm) size of magnetic iron oxide nanoparticles, homogeneous coverage of HNT by magnetic nanoparticles was achieved. Magnetic measurements confirmed that the incorporation of MHNT into PCL/Gel nanofibers led to the preparation of superparamagnetic nanofibers with a saturation magnetization of 0.67 emu/g with 12 wt.% MHNT. Moreover, after the application of MHNT, an improvement in nanofiber structure and an important reduction in fiber diameter was observed. Unlike fibers based on HNT, the MHNT-containing fibers were uniform and without serious defects. In addition, through the application of both HNT and MHNT in PCL/Gel nanofibers, a significant (>100%) improvement in the reinforcing effect was observed. The highest improvement was observed for nanofibers containing 9 wt.% MHNT when increases in tensile strength reached almost 300%, while the elongation at break reached a five-fold improvement. In summary, the prepared PCL/Gel/MHNT nanofibers are multifunctional, softmagnetic, environmentally friendly, biodegradable, high-strength, highly elastic materials suitable for new progressive biomedical applications.

## Figures and Tables

**Table 1:** EDX analysis of HNT, MHNT, and PCL/Gel/MHNT nanofibers (average values, the table does not show other minor elements such as Na, Ca, Cl, Mg, and K, which were also detected).

wt.%	C	N	O	Al	Si	Fe
HNT	0.0	0.0	64.9	17.6	15.5	<0.3
MHNT	0.0	0.0	64.0	14.9	13.2	8.2
PCL/Gel/MHNT (6–12 wt.%)	56.3	16.8	24.8	0.6	0.5	0.3–0.5

**Table 2:** Summary of magnetic properties of magnetized HNTs and prepared nanofibers at 300 K. In brackets are values for MHNT measured at 2 K.

Sample	M_S_ (emu/g)	M_R_ (emu/g)	H_C_ (Oe)	T_B_ (K)
MHNT	7.8 (9.6)	0.22 (2.5)	16 (216)	120 K
6 wt.%	0.27	0.009	14	-
9 wt.%	0.6	0.019	15	-
12 wt.%	0.67	0.021	15	-

## Data Availability

The data presented in this study are available upon request from the corresponding author.
